# Storage Stability of Kinnow Fruit (*Citrus reticulata*) as Affected by CMC and Guar Gum-Based Silver Nanoparticle Coatings

**DOI:** 10.3390/molecules201219870

**Published:** 2015-12-18

**Authors:** Syed Wasim Ahmad Shah, Muhammad Jahangir, Muhammad Qaisar, Sher Aslam Khan, Talat Mahmood, Muhammad Saeed, Abid Farid, Muhammad Liaquat

**Affiliations:** 1 Section of Food Science and Technology, Department of Agricultural Sciences, University of Haripur, Haripur 22620, KPK, Pakistan; syedhisyed@hotmail.com (S.W.A.S.); mj_awan@hotmail.com (M.J.); talat204@hotmail.com (T.M.); 2 Pakistan Council of Scientific and Industrial Research (PCSIR) Laboratories Complex, Peshawar 22620, KPK, Pakistan; fqkhan65@hotmail.com; 3 Section of Plant Breeding and Genetics, Department of Agricultural Sciences, University of Haripur, Haripur 22620, KPK, Pakistan; sheraslamqau@gmail.com; 4 Section of Entomology, Department of Agricultural Sciences, University of Haripur, Haripur 22620, KPK, Pakistan; pest_mgt@yahoo.com (M.S.); Abidfarid786@gmail.com (A.F.)

**Keywords:** kinnow, silver nanoparticles, CMC, guargum, ascorbic acid, antioxidant activity

## Abstract

The influence of carboxy methyl cellulose (CMC) and guargum-based coatings containing silver nanoparticles was studied on the postharvest storage stability of the kinnow mandarin (*Citrus reticulata* cv. Blanco) for a period of 120 days (85%–90% relative humidity) at 4 °C and 10 °C. Physicochemical and microbiological qualities were monitored after every 15 days of storage. Overall results revealed an increase in total soluble solid (TSS), total sugars, reducing sugars and weight loss but this increase was comparatively less significant in coated fruits stored at 4 °C. Ascorbic acid, total phenolics, and antioxidant activity was significantly enhanced in coated fruits stored at 4 °C. Titratable acidity significantly decreased during storage except for coated kinnow stored at 4 °C. In control samples stored at 10 °C, high intensity of fruit rotting and no chilling injury was observed. Total aerobic psychrotrophic bacteria and yeast and molds were noticed in all treatments during storage but the growth was not significant in coated fruits at 4 °C. Kinnow fruit can be kept in good quality after coating for four months at 4 °C and for 2 months at 10 °C.

## 1. Introduction

Kinnow (*Citrus reticulate* cv. Blanco) is a fruit in the citrus family. Kinnow, grown in Pakistan, is a cross between the King Orange (*Citrus nobilis*) and Willow Leaf Orange (*Citrus deliciosa*) of riverside California [1]. Mandarins are cherished around the globe due to their nutritional value, physicochemical properties, natural antioxidants, and sensory attributes [2]. The natural polyphenols in oranges comprise some bioactive compounds like hesperidins, vitamin C, carotenoid, naringin, ferulic acid, hydrocinnamic acid, and cyaniding glucoside [3]. These plant metabolites are effective in boosting the immune system against coronary heart diseases, cancer, and various infections, and also aidthe absorption of iron and zinc [4].

Poor postharvest practices result in loss of quality through the development of physiological disorders like weight loss, pathological disorder through microbial attack, and fruit rot [5]. These losses, which ranged between 35% and 40% in Pakistan [6], can be minimized by several technologies like low temperature storage, wax coating, and modified atmospheric storage [7]. Among these various techniques, food coating has gained great interest for fresh and minimally processed fruits and vegetables not only due to the possibility of keeping certain quality characteristics (texture, firmness, and dehydration) but also because coatings can carry useful additives like antimicrobial and anti-browning agents [8].

Mandarins can be kept in good quality under ambient storage conditions for only 15–30 days and have the shortest storage life among all the fruits of the citrus family. To extend the storage life of mandarins, low temperature storage (5–8 °C) combined with high relative humidity (90%–95%) is desirable [9]; storage at 5 °C and 90%–95% relative humidity is also acceptable [10]. Storage of mandarins after coating at relatively high humidity in cold storage is believed to be highly beneficial in preserving fruit quality [11].

Most suitable temperature of 4 ^o^C for extending shelf life was already demonstrated for fresh cut carrots coated with calcium-alginate coating mixed with silver-montmorillonite [12]; for wax coated mandarin [11,13]. However, 2–8 °C at 85%–95% relative humidity was also highly acceptable for preserving mandarin quality after wax emulsion coating [14]; 2–10 °C at 90%–95% relative humidity was also adapted for mandarin storage [15] and for freshly cut silver nanoparticles-PVP-coated asparagus storage [16].

Nanotechnology is being utilized for extension of shelf life of fresh commodities [17,18]. Nanoparticles are commonly described as solid, colloidal particles that are in the range of 10–100 nm [19]. Nanoparticles of below 100 nm are considered to have excellent antimicrobial activity [20]. Among the noble metals, silver nanoparticles have been receiving the most attention due to their preservation activities in food processing applications [16,18].

Silver ions have been demonstrated to be an excellent antimicrobial agent against *E. coli* [21], psychrotrophic bacteria, and molds [16]. These particles destroy microbes by damaging their cell membrane and even disintegrating DNA after penetration in the cell [20]. The safety limit of silver declared by EU safety regulations for foods is 0.05 mg/kg [21]. Therefore, the use of edible coatings as a carrier of silver nanoparticles to foods is a novel approach [22]. It is proved that a silver concentration of 0.06 mg·L^−1^ is acceptable for coating fruits and vegetables [16].

To our knowledge, there is not yet any published study on the use of CMC and guargum-based silver nanoparticle coating for preservation and extension of the storage life of fruits. Therefore, the objective of this study was to investigate the effects of CMC and guargum-based silver nanoparticle coating in prolonging the shelf life of the kinnow mandarin.

## 2. Results and Discussion

### 2.1. Physicochemical Evaluation

#### 2.1.1. Weight Loss and Chilling Injury

**Figure 1:**
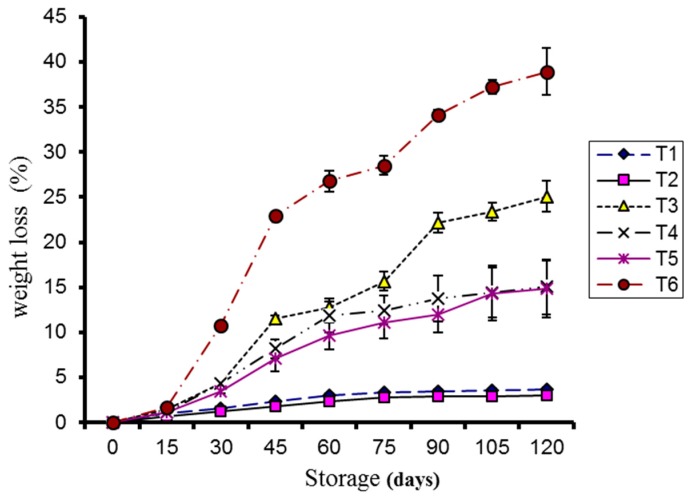
Effect of CMC and guargum-based silver nanoparticle coatings on the weight loss of kinnow fruit stored at 4 and 10 °C. The vertical bars represent standard error of the means. Values represented as mean ± SE of three replicates.

The data showed that coatings markedly reduced weight loss in the kinnow fruit during storage, particularly at 4 °C, compared to the control. Weight loss was significantly increased (*p* < 0.05) during storage in all treatments except in the coated kinnow stored at 4 °C (Figure 1). Maximum weight loss was observed in control samples stored at 10 °C and 4 °C, respectively.

**Figure 2:**
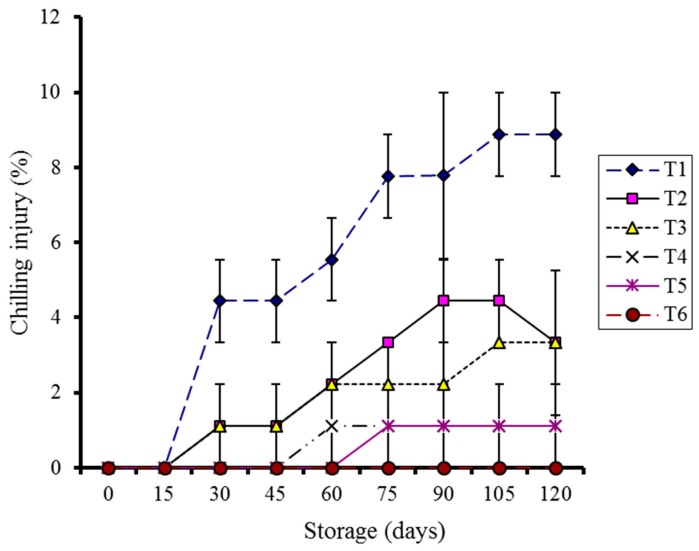
Effect of CMC and guargum-based silver nanoparticle coatings on the chilling injury of kinnow fruit stored at 4 and 10 °C during storage. The vertical bars represent standard error of the means. Values represented as mean ± SE of three replicates.

Chilling injury (CI) was significantly affected (*p* < 0.05) during storage in all the samples stored at 4 °C during storage. The effect of storage time and temperature on CI remained non-significant during storage in kinnows kept at 10 °C, whether they were coated or not (Figure 2). Control samples stored at 4 °C showed a similar CI (3.33%) to guargum-Ag coated kinnow at the end of storage. However, the control showed higher weight loss, fruit rot, and microbial count during storage. The data showed that kinnows stored at 10 °C were the least affected by CI but showed a high intensity of rotting.

#### 2.1.2. Total Soluble Solids and Titratable Acidity

**Figure 3:**
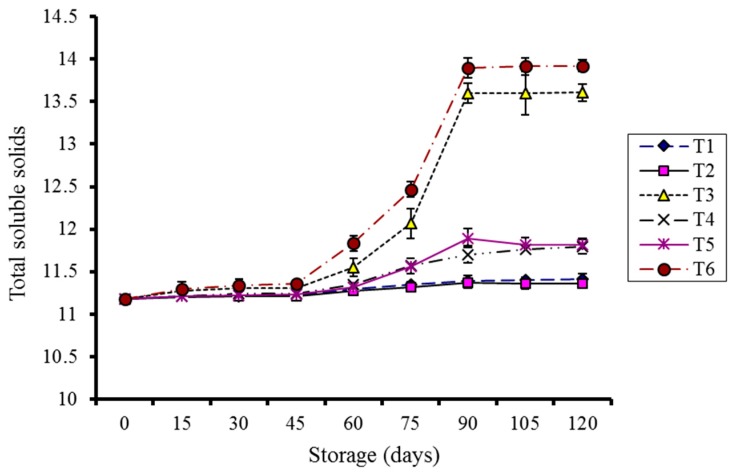
Effect of CMC and guargum-based silver nanoparticle coatings on the total soluble solids of kinnow fruit stored at 4 and 10 °C during storage. The vertical bars represent standard error of the means. Values represented as mean ± SE of three replicates.

In general, there was a gradual increase in total soluble solids (TSS) during storage (Figure 3). Significant variance (*p* < 0.05) was observed among the storage days, treatments, and storage temperatures. Control kinnows stored at 10 °C and 4 °C showed significantly higher TSS as compared to coated treatments.

**Figure 4:**
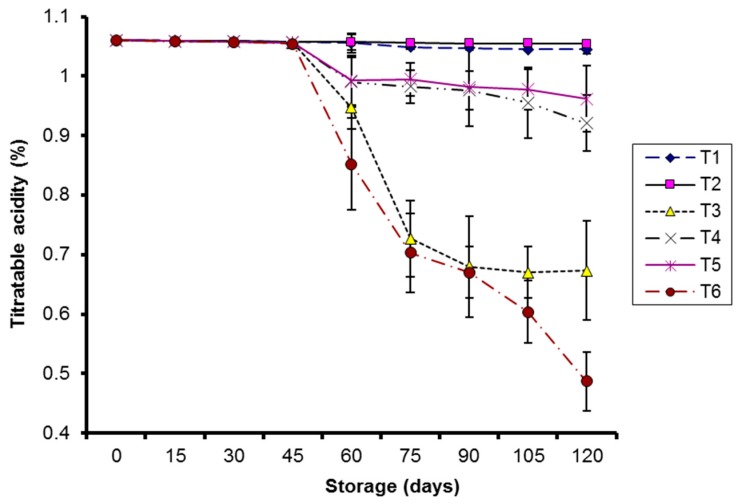
Effect of CMC and guargum-based silver nanoparticle coatings on the titratable acidity of kinnow fruit stored at 4 and 10 °C during storage. The vertical bars represent standard error of the means. Values represented as mean ± SE of three replicates.

A significant effect (*p* < 0.05) on titratable acidity (TA) of kinnow was observed in coated fruits at 10 °C and in control samples at both the storage temperatures. The effect remained non-significant (*p* > 0.05) in coated fruits stored at 4 °C during storage. TA decreased significantly in control samples at both storage temperatures (Figure 4).

#### 2.1.3. Total Sugars and Reducing Sugars

**Figure 5:**
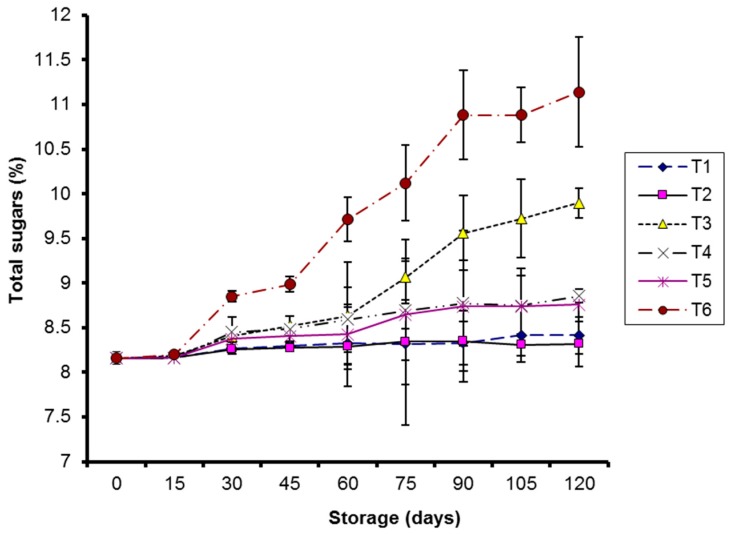
Effect of CMC and guargum-based silver nanoparticle coatings on the total sugars of kinnow fruit stored at 4 and 10 °C during storage. The vertical bars represent standard error of the means. Values represented as mean ± SE of three replicates.

Total sugars (TS) significantly increased (*p* < 0.05) in all treatments during storage, irrespective of storage temperatures. Control and coated samples stored at 10 °C showed significantly higher TS as compared to coated fruits stored at 4 °C on 120th day of storage (Figure 5).

**Figure 6:**
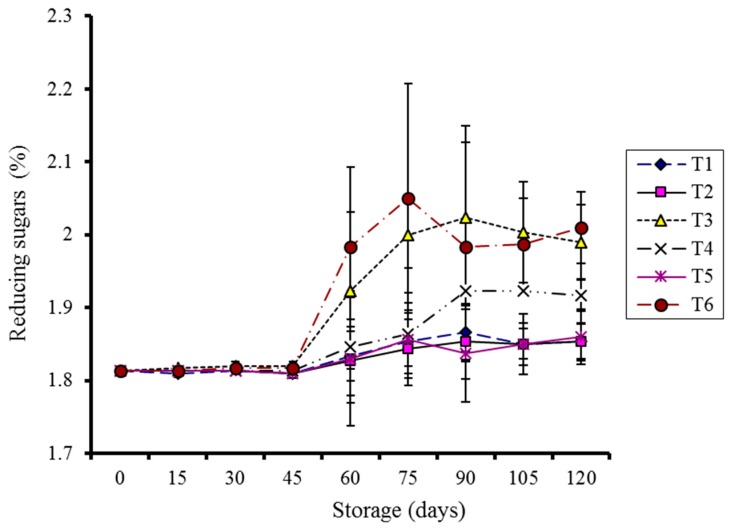
Effect of CMC and guargum-based silver nanoparticle coatings on the reducing sugars of kinnow fruit stored at 4 and 10 °C during storage. The vertical bars represent standard error of the means. Values represented as mean ± SE of three replicates.

Gradual increase in reducing sugars (RS) was observed during storage in all the treatments, irrespective of coating application (Figure 6). Non-significant variance (*p* > 0.05) was observed among the storage days, different treatments, and storage temperatures for coated samples while the control demonstrated a significant effect (*p* < 0.05).

#### 2.1.4. Ascorbic Acid

**Figure 7:**
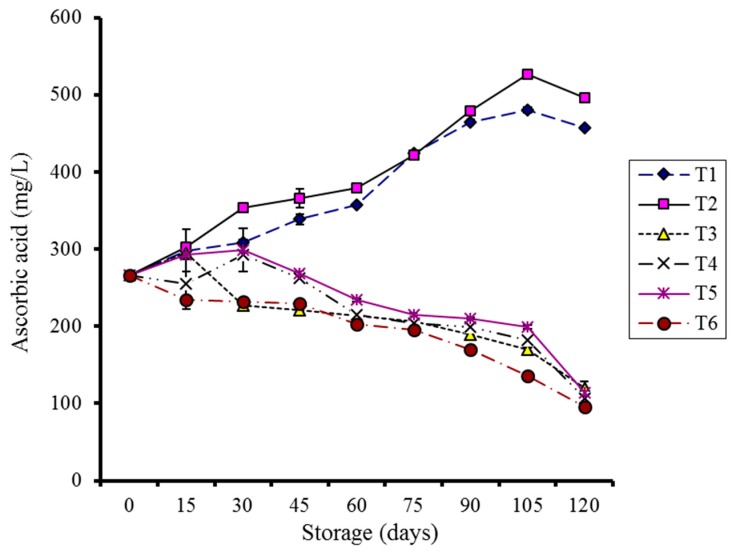
Effect of CMC and guargum-based silver nanoparticle coatings on the ascorbic acid of kinnow fruit stored at 4 and 10 °C during storage. The vertical bars represent standard error of the means. Values represented as mean ± SE of three replicates.

Ascorbic acid (AA) was significantly enhanced during storage in coated fruits at 4 °C, as illustrated in Figure 7. An initial increase in AA was also observed in coated fruits stored at 10 °C for 30 days and in the control at 4 °C for 15 days. Control samples stored at 10 °C showed a consistent decrease. A significant correlation (*p* < 0.05) between storage, treatments, and temperatures was noted.

#### 2.1.5. Total Phenolic Contents

**Figure 8:**
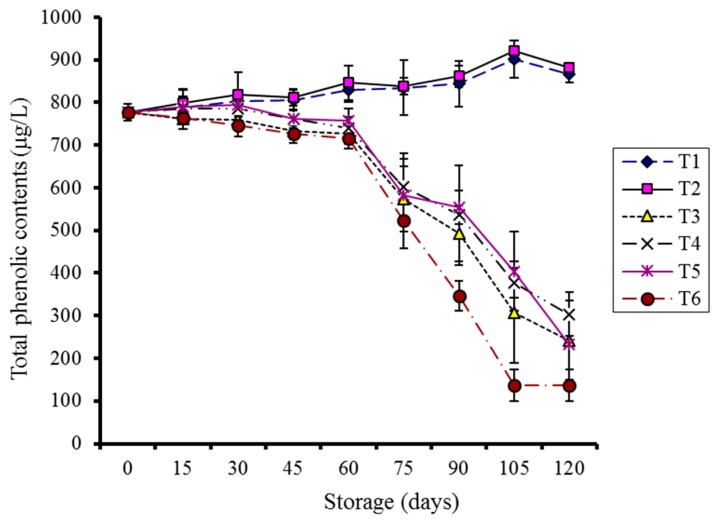
Effect of CMC and guargum-based silver nanoparticle coatings on the total phenolic contents of kinnow fruit stored at 4 and 10 °C during storage. The vertical bars represent standard error of the means. Values represented as mean ± SE of three replicates.

The relationship between storage, treatments, and temperatures was observed to be significant (*p* < 0.05) during storage. Total phenolic contents increased during storage for coated kinnow fruits kept at 4 °C until the 105th day and then started decreasing. Coated fruits kept at 10 °C shown a small increase during the first 30 days of storage, followed by a sharp decline. To the contrary, the control samples showed a continuous decrease (Figure 8).

#### 2.1.6. Antioxidant Activity

**Figure 9:**
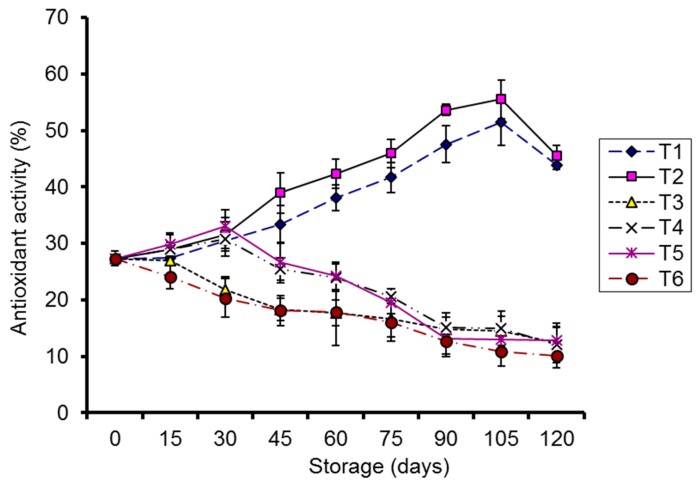
Effect of CMC and guargum-based silver nanoparticle coatings on the antioxidant activity of kinnow fruit stored at 4 and 10 °C during storage. The vertical bars represent standard error of the means. Values represented as mean ± SE of three replicates.

A significant (*p* < 0.05) correlation was observed between the storage and antioxidant activity at both storage temperatures. The antioxidant activity of the kinnow fruits treated with CMC-Ag and a guargum-Ag coating stored at 4 °C was significantly enhanced during storage. To the contrary, control samples stored at 4 °C and 10 °C exhibited a continual decrease. An increasing trend for the first 30 days of storage, followed by a continual decrease, was noted in coated kinnows stored at 10 °C (Figure 9).

### 2.2. Microbiological Evaluations

#### 2.2.1. Fruit Rotting

**Figure 10:**
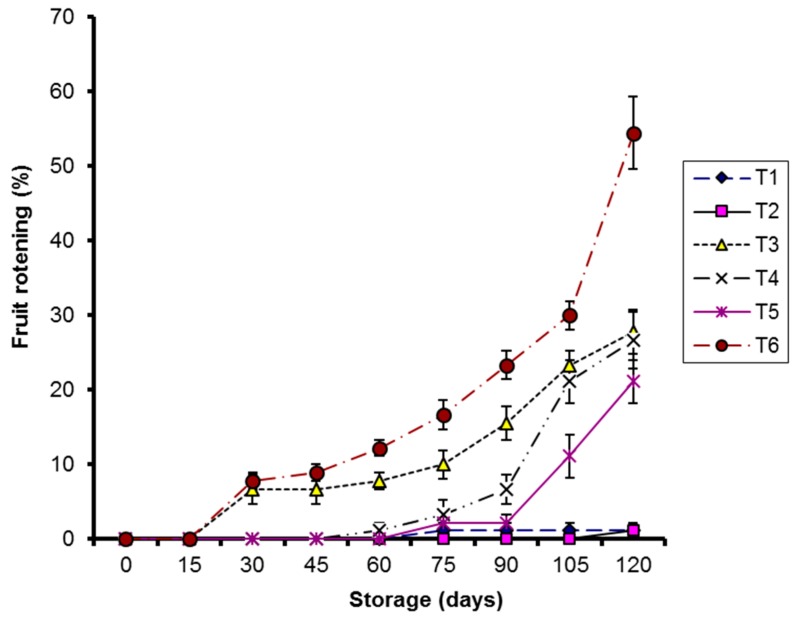
Effect of CMC and guargum-based silver nanoparticle coatings on the rotting of kinnow fruit stored at 4 and 10 °C during storage. The vertical bars represent standard error of the means. Values represented as mean ± SE of three replicates.

A significant correlation (*p* < 0.05) was observed between treatments and storage for coated kinnows at 10 °C storage and the control, while a non-significant correlation (*p* > 0.05) was noted for CMC-Ag- and guargum-Ag-coated treatments stored at 4 °C (Figure 10). Control samples stored at 10 °C were most affected during storage.

#### 2.2.2. Aerobic Psychrotrophic Count, Yeast, and Mold Count

**Figure 11:**
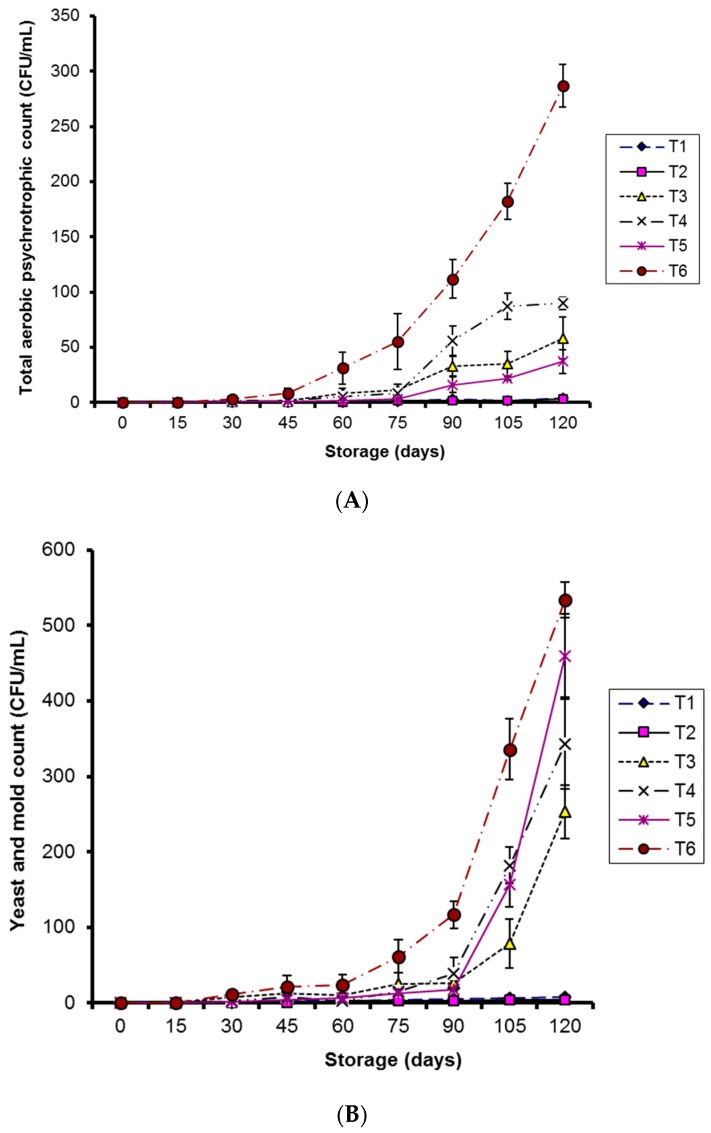
(**A**) Effect of CMC and guargum-based silver nanoparticle coatings on the total aerobic psychrotrophic count in kinnow fruit stored at 4 and 10 °C during storage. The vertical bars represent standard error of the means. Values represented as mean ± SE of three replicates; (**B**) Effect of CMC and guargum-based silver nanoparticle coatings on the yeast and mold count in kinnow fruit stored at 4 and 10 °C during storage. The vertical bars represent standard error of the means. Values represented as mean ± SE of three replicates.

Both CMC-Ag and guargum-Ag coatings significantly reduced (*p* < 0.05) the increase in total aerobic psychrotrophic count and yeast and mold count in kinnows stored at 4 °C compared to other treatments during storage (Figure 11A,B). Control samples at 10 °C storage were most affected during storage.

Figure 11B demonstrates that control samples were seriously affected by yeast and molds during storage. CMC-Ag- and guargum-Ag-coated kinnows exhibited good quality at 4 °C.

### 2.3. Metal Ion Releasing Measurement

The quantity of silver in the squeezed orange juice after 120 days of storage found 0 ppm in all the treatments, whether coated or not. The atomic absorption spectrophotometer data confirmed that leaching of silver in the fruit matrix did not happen during storage; it remained on the peel.

### 2.4. Post-Storage Release Test

Judges observed no sign of chilling injury affecting quality in CMC-Ag- and guar gum-Ag-coated kinnow for a week of storage. No sign of chilling injury was apparent within a week except a slight drying of the kinnow surface after the seventh day of storage at ambient temperature. Fruit rotting was also not observed until after a week of storage.

### 2.5. Discussion

The experimental data confirmed that both CMC-Ag and guargum-Ag coatings markedly reduced the weight loss in those kinnow mandarin treatments stored at 4 °C. Weight loss in fresh fruits and vegetables has not only been associated with vapor pressure at various locations, but also occurs through respiration phenomenon [23]. Weight loss in uncoated kinnow can be above 33%, rendering fruit unacceptable for consumption [24].

Temperatures lower than 10 °C can influence mandarins, with chilling injury rendering fruit aesthetically undesirable for consumption [25]; below 4 °C mandarins encounter chilling injury [26]. The hybrid nature of mandarins is believed to be the major reason behind their sensitivity to chilling injury. To extend shelf life, storage at low temperature coupled with high relative humidity is desirable [27]. The coated kinnow showed excellent postharvest storage stability at 4 °C but demonstrated high chilling injury. This suggests that for the fruits to tolerate the combined stress imposed by a silver nanoparticles coating and low-temperature storage (4 °C), they have started production of primary and secondary metabolites like sugars, phenolics, and ascorbic acid. Postharvest conditions are the potential stress to plants that may shift the plant’s response towards the defensive side by producing primary and secondary metabolites [28]. Thus, chilling injury enhances the synthesis of antioxidants like ascorbate and glutathione [29].

Total soluble solids (TSS) were significantly increased in uncoated kinnows compared to coated, which might be due to the barrier provided by coating against respiration and evaporation. Higher TSS in oranges has been related to an increased rate of respiration and evaporation from the fruit’s surface [24].

Coating application at 4 °C did not greatly affect the titratable acidity (TA). The data showed less of a decrease in TA in coated kinnows compared to uncoated, probably due to the slow rate of respiration and metabolic processes converting citric acid into sugars as a function of applied coatings. Citric acid and isocitric acids were reported to be the major compounds found to contribute the acidity in mandarins [30].

An increase in total sugars was reported due to the enzymatic breakdown of complex carbohydrates of the cell wall structure into simpler sugars during storage; this was also due to increased respiration at ambient storage temperatures [31]. An increase in reducing sugars during storage has been associated with starch breakdown into simpler sugars [32].

The increasing trend in ascorbic acid contents in coated kinnows stored at 4 °C supports the hypothesis that these fruits, under the continuous abiotic stress imposed by silver nanoparticles coating and low-temperature storage (4 °C), may have started producing primary and secondary metabolites and regenerating ascorbic acid, while other treatments exhibited catabolism. The slow decrease in acidity and lower increase in reducing sugars confirms that the increased sugars may have started an alternate pathway of ascorbic acid biosynthesis [33,34,35]. Low-temperature exposure of broccoli was reported to enhance the ascorbic acid contents [36]. This increase might be due to an indirect activation of ascorbic acid biosynthesis by the production of reducing and non-reducing sugars [28]. The release of d-glucose, l-galactose, and l-galacturonic acid acts as a precursor to ascorbic acid, helping to maintain or even increase ascorbic acid contents [33]. An increase of ascorbic acid was previously reported for the effect of salicylic acid on kiwi fruit [37] and navel oranges [38] during storage.

The present study suggests that coating plus low-temperature storage (4 °C) have geared the production of phenolics. These compounds are influenced by the climate as well as biotic and abiotic stress during pre-and postharvest conditions [39]. Low temperature has also been reported to contribute in preserving phenolics in plants and is often associated with an increase of polyphenols due to stress [40].

These results of antioxidant activity also demonstrated that silver nanoparticle coatings, in combination with low-temperature storage, stimulated the scavenging capacity of the kinnow fruit. The decrease in antioxidant activity of coated kinnows stored at 4 °C after 105 days was probably due to intensive degradation of ascorbic acid and phenolics through biosynthesis. Antioxidant activity has been reported to depend upon ascorbic acid, total phenols, anthocyanins, and flavonoids [41]. Our results on antioxidant activity support the aforementioned findings on ascorbic acid and total phenolic contents.

A study of fruit rot clearly showed that silver nanoparticle coatings had markedly reduced fruit rot due to the strong toxicity imposed by silver nanoparticles against a wide range of microorganisms, as already described [42].

Psychrotrophic bacteria and molds represent the predominant microflora influencing the shelf life of fruits and vegetables during low-temperature storage [43]. The total aerobic psychrotrophic count during the entire storage period remained below 10^4^ CFU/mL in all the treatments, which is also the set threshold level for such microorganisms [44]. Very low aerobic psychrotrophic count in coated fruit at 4 °C storage is due to the strong toxicity of silver ions against a variety of microbes, as has also been described [42]. Reduction in total aerobic psychrotrophic count influenced by silver ions has previously been noted for freshly cut asparagus [16] and fresh orange juice [42].

Our results demonstrated that yeast and molds were more adapted to kinnow mandarins during cold storage than bacteria, as already declared for citrus fruits [42]. The shelf life of cold-stored oranges depends mainly on yeast and mold growth [45]. 

Silver was not detected in all the treatments, which suggested that leaching of silver into the fruit did not happen and fruit remained consumable after a prolonged storage of 120 days, though the added quantity of silver in the coatings was also in the acceptable range for consumers. The optimum concentration of silver is 0.04 mg/Kg for the preservation of vegetable juice [46].

The results of post-storage release tests indicated that CMC-Ag- and guargum-Ag-coated kinnows might not show existing symptoms of chilling injury in the future but rather dehydrate rapidly after a week probably due to a sudden intense change of storage environment, particularly exposure to high temperature and low relative humidity. Navel oranges stored at 20 °C and shifted from 45% relative humidity to 95% showed a high rate of chilling injury symptoms compared to very low signs if shifted from 95% to 45% relative humidity [47]. Therefore, it was concluded that the chilling injury evident during storage did not affect the market quality of kinnows kept at 20 °C for seven days.

## 3. Experimental Section

### 3.1. Preparation of Coating Base

A coating emulsions base was prepared by a slight modification of a previously reported method [32]. Beeswax (1 g), 50 g olive oil, 10 g lecithin, and 200 mL of 0.85% saline water (0.85 g AR grade NaCl dissolved in 100 mL distilled water) were combined in a beaker and stirred well. One hundred fifty milliliters of a solution of CMC (CPKelco-CEKOL, Äänekoski, Finland) and guargum (Hebgen Guargum, Karachi, Pakistan) at 3% was added separately for each coating emulsion base and shaken vigorously for emulsion formation. The volume of each coating base was made 1000 mL using 0.85% saline water after the addition of 800 ppm sodium hypochlorite (UNI-Chem, Wuhan, China).

### 3.2. Preparation and Characterization of Silver Nanoparticles

**Figure 12:**
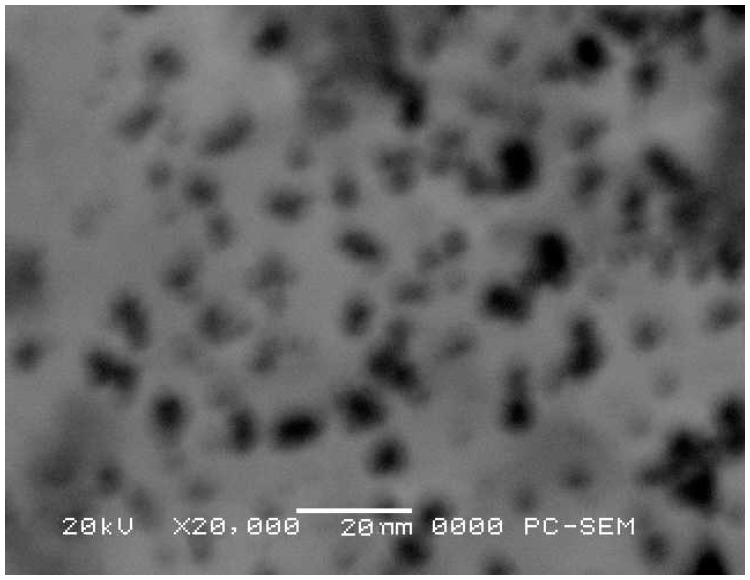
Scanning electron microscopy (SEM) of silver nanoparticles (×20,000).

Silver nanoparticles were prepared by the chemical reduction method, as previously reported [16]. Silver nitrate (0.1 M AgNO_3_, 99.9% Merck, Darmstadt, Germany), 0.01 M sodium borohydride (NaBH_4_, DAEJUNG chemicals, Shiheung-city, Korea), 0.01 M polyvinylpyrrolidone (PVP) (bioPLUS Fine Research Chemicals, Dublin, OH, USA), and 1.5% glycerol (Shanghai Chemical Reagent Company, Shanghai, China) were mixed with a final practical silver concentration of 0.06 mg·L^−1^ (silver nanoparticles diameter 20 nm), which is safe for consumers [16]. Mixed at a 1:1 ratio were 0.01 M sodium borohydride and 0.01 M polyvinylpyrrolidone (PVP). To this mixture, 0.1 M Silver nitrate was added drop by drop till development of a brown color, which indicates the formation of silver nanoparticles. Glycerol was added (1.5/100 mL) and the silver concentration was measured to be 0.06 mg·L^−1^ in a prepared silver nanoparticle coating using an atomic absorption spectrophotometer at 328.1 nm [48]. The micrograph of a scanning electron microscope (JSM-6460LV-Tokyo, Japan) has confirmed that the diameter of silver nanoparticles prepared in this research was about 20 nm. Silver nanoparticles were almost spherical, with a mean diameter of about 10–25 nm (Figure 12).

### 3.3. Preparation of CMC and Guargum Based Silver Nanoparticles Coatings

Both the above emulsions (3.1 and 3.2) were mixed in a 1:1 ratio separately for a CMC-based silver nanoparticle coating (CMC-Ag coating) and a guargum-based silver nanoparticle coating (guargum-Ag coating). The final practical silver concentration of 0.03 mg·L^−1^ used for coating was confirmed by atomic absorption spectrophotometer at 328.1 nm [48]. These emulsions with PVP, CMC, guargum, oil, and beeswax formed a thin coating on the kinnow peel surface when the water evaporated, leaving silver nanoparticles evenly distributed in the coating matrix.

### 3.4. Plant Material and Handling

Freshly harvested kinnow (*Citrus reticulata* cv. Blanco) fruits were obtained from a commercial farm in Kot Momin, District Sargodha, Pakistan (Supreme Fruit Farms). Fruits were picked 3–4 h before sunset, immediately analyzed for total soluble solids (TSS) (^o^brix) and titratable acidity (% citric acid) in an on-site temporary farm laboratory, and brought to the laboratory for coating and storage within 4 h after harvest. Mature, evenly sized, bright red kinnows were used in the study. All kinnows were thoroughly washed in a 100 mg·L^−1^ sodium hypochlorite solution for 15 min at room temperature and then kept at ambient temperature to remove surface liquid. Fruits were immersed in the CMC-Ag and guargum-Ag coating separately for 3 min [32]. Non-coated fruits were treated as the control. All the kinnow mandarin samples were stored in board cartons (286 × 220 × 150 mm) with a small hole at each of the four sides for up to 120 days at 4 °C and 10 °C at 85%–95% relative humidity. Both the control and coated samples were analyzed at 15 day intervals from the day of coating. All experiments were performed in triplicate for different physicochemical and microbiological parameters and the average of the three results was evaluated statistically. Various treatments were identified as: T1—kinnow coated with CMC-Ag coating stored at 4 °C; T2—kinnow coated with guargum-Ag coating stored at 4 °C; T3—control stored at 4 °C; T4—kinnow coated with CMC-Ag coating stored at 10 °C; T5—kinnow coated with guargum-Ag coating stored at 10 °C; T6—control stored at 10 °C.

### 3.5. Physicochemical Evaluation

#### 3.5.1. Weight Loss and Chilling Injury

Percent weight loss was measured using a digital weighing balance (AY 220 Shimadzu Corporation, Kyoto, Japan), as reported in the standard method [49]. A total of 90 kinnow were used for each treatment in triplicate. 

The incidence of chilling injury on kinnow fruit was determined after a slight modification of the reported method [14,50], by counting the total number of fruits showing chilling injury symptoms such as irregular brown lesions, yellow water-soaked spots, or even sunken areas on the fruit peel. A total of 90 kinnow were used for each treatment in triplicate during storage. The threshold level for severity of chilling injury was set as 10% of the surface area of kinnow affected.

#### 3.5.2. Total Soluble Solids and Titratable Acidity

Total soluble solids (TSS) were determined using a Brixby hand refractometer (ATAGO, SR-500, Tokyo, Japan), as already described in the standard method [49]. Fresh juice was squeezed in triplicate for analysis during storage.

Titratable acidity (TA) was determined (g citric acid 100 mL^−1^), as previously reported in the standard method [49]. Fresh kinnow juice was used for each treatment in triplicate for analysis during storage.

#### 3.5.3. Total Sugars and Reducing Sugars

Total sugars and reducing sugars were calculated by the Lane and Eynon method, as described in the previously reported standard method [49]. Freshly squeezed juice in triplicate was used for analysis during storage for each treatment.

#### 3.5.4. Ascorbic Acid

Ascorbic acid (AA) was determined by the HPLC technique (HPLC with UV detector, LC-8A Shimadzu) through slight modification of the previously reported method [51,52]. Kinnows were randomly picked and divided into three samples containing three fruits for each treatment. Fresh juice was extracted in triplicate for analysis. Kinnow juice was squeezed using a citrus juice extractor (BRAUN Gmbh, Pfungstadt, Germany), filtered through a double layer of cheese cloth, and centrifuged at 7000 rpm for 10 min at 4 °C. The extract was filtered through a 0.22-µm membrane filter; the aliquot was again filtered through a 0.45 µm syringe membrane filter (Sartorius, Tokyo, Japan). An aliquot (50 µL) was injected in HPLC for chromatography analysis with a Shimadzu C-18 column (15 cm × 4.6 mm, pore size 5 µm) coupled with a HyperODS guard column at 245 nm UV detector with an acetonitrile-phosphate buffer (pH: 2.6) as the mobile phase (1.5 mL/min). AA concentration (mg/L) was determined by comparison of UV spectrum and retention time with reference standards of known concentration (99.9% l-ascorbic acid analytical standard, Supelco, Munich, Germany).

#### 3.5.5. Total Phenolic Contents

Total phenolic contents were measured by the Folin-Ciocalteu reagent method, as previously described [53,54]. Fresh kinnow juice was squeezed in triplicate for analysis, filtered through a double layer of cheese cloth, and centrifuged at 7000 rpm at 4 °C for 10 min after addition of solvent (50 mL methanol/100 mL juice). Half a milliliter of extract was mixed with 2.5 mL distilled water, 0.5 mL (1:1). Folin-Ciocalteu reagent was added (99.9% Merck) and the resulting mixture was incubated for 3 min at 25 °C. To each tube, 2 mL of 20% sodium carbonate (99%, BDH, Sutton Coldfield, UK) was added and kept for 1 min. Absorbance was noted at 650 nm on spectrophotometer using gallic acid standard gradient curve. Total phenolic contents were determined as µg/mL gallic acid equivalents (GAE)/mL.

#### 3.5.6. Antioxidant Activity

Antioxidant activity was determined by slight modification of DPPH free radical scavenging method [55]. Freshly squeezed juice was used for analysis in triplicate during storage, filtered, and centrifuged at 7000 rpm for 10 min after addition of solvent (methanol 50 mL/100 mL juice). One milliliter (0.5 mM) of DPPH (99.9%, Sigma Aldrich, Steinheim, Germany) was added to 2 mL fruit extract. To this mixture, 2 mL of distilled water was added and left to stand for 30 min at room temperature. The absorbance was measured at 517 nm after every 1 min for 3 min using UV-Vis spectrophotometer. The absorbance of DPPH in the control was also noted. Antioxidant activity was calculated as the percent change in absorbance compared to the control for 3 min, corresponding to the percentage of DPPH scavenged [53].

Antioxidant activity (%) = Test sample absorbance/Control absorbance × 100
(1)

### 3.6. Microbial Evaluations

#### 3.6.1. Fruit Rot

Kinnows showing greenish-gray mold spots on the fruit surface were counted for each treatment and the percentage of fruit rotting was calculated as already described [2]. The threshold level for severity was set as 8% of fruits affected during storage, indicating the end of shelf life. A total of 90 kinnows per treatment were used in triplicate for the study.

Fruit rotting (%) = Number of affected fruits/Number of fruits in treatment × 100
(2)

#### 3.6.2. Aerobic Psychrotrophic Count, Yeast, and Mold Count

The samples were analyzed for total aerobic psychrotrophiles and yeast and mold count. Kinnow juice was taken from each treatment in triplicate by injecting the needle of a sterile disposable syringe (1cc) into the fruit, inoculated directly in prepared plates by the pour plate method, as already described [42], and colonies were counted as CFU/mL using colony counter (Stiart-SC6 plus, Bibbly Scientific, Staffordshire, UK). The samples were inoculated on plates containing media as follows: (1) plate count agar (Merck VM291763 122) for total aerobic psychrotrophic count, incubated at 30 °C for 72 h; (2) Sabouraud 4% dextrose agar (Merck VM207838 046) for yeast and mold count; petri dishes were incubated at 25 °C for 120 h.

### 3.7. Metal Ion Releasing Measurement

Silver leached into fruit during storage was determined by the standard method [48] using an atomic absorption spectrophotometer (Hitachi Z-2000, Tokyo, Japan) at 328.1 nm.

### 3.8. Post-Storage Release Test 

CMC-Ag- and guargum-Ag-coated kinnows after successful completions of 120 days of storage were assessed for market acceptance by slight modifications of the reported method [15]. These fruits, stored at 4 °C, were shifted to the ambient temperature (20 °C) for 7 days after 120 days of storage, mainly to assess how chilling injury affects the quality of kinnows when they arkeptat 20 °C on shopkeepers’ shelves. Chilling injury (%) symptoms affecting quality and fruit rot (%) were assessed daily for a week. A scale of 1 to 4 was used for chilling injury with 1 = healthy; 2 = slightly affected; 3 = moderately affected; and 4 = severely affected. The scale depends on the extent of damage introduced by chilling injury to fruit quality. Additionally, a panel of five experienced judges was also invited to assess the chilling injury affecting fruit quality for a week of storage. Judges were given a form with a hedonic scale (1–4) to fill out. Fruit rot was assessed by applying strict threshold level of 2% fruits affected as the end of storage life. One hundred kinnows of both treatments were selected for daily inspection.

### 3.9. Statistical Analysis

The data obtained were statistically analyzed for Analysis of Variance (ANOVA) using two-factorial Completely Randomized Design (CRD) and Duncan’s Multiple Range Test (DMRT), as already reported [56]. Degree of significance was calculated by least significant difference at *p* < 0.05. The contrast between different treatments was found by applying the Pearson correlation coefficient technique using SPSS software (New York, NY, USA).

## 4. Conclusions

Two new coatings were found to improve the shelf life of fresh kinnow fruit. The results demonstrated that CMC-Ag and guargum-Ag coatings can be best utilized to control dehydration and retard microbial growth in kinnows. The antimicrobial properties of CMC-Ag and guargum-Ag coatings were also manifest in the obtained results. The experimental results demonstrated that the combined use of a silver nanoparticle coating along with low-temperature storage (4 °C) maintained excellent preservation of the kinnow mandarin, thus prolonging the shelf life to about 120 days compared to 60 days for coated kinnows stored at 10 °C. The present study suggests that CMC and guargum-based silver nanoparticle coatings, in combination with low-temperature storage (4 °C), might be an efficient strategy for improving ascorbic acid, total phenolics, and antioxidant activity. Further studies should be conducted for metabolomic evaluation to give a detailed metabolomic picture for understanding the mechanism of action for CMC-Ag coating and guargum-Ag coating on cellular response during low-temperature storage.
